# Lattice Models in Molecular Thermodynamics: Merging the Configurational and Translational Entropies

**DOI:** 10.1021/acs.jpcb.4c06473

**Published:** 2024-12-13

**Authors:** Per-Olof Åstrand, Rodrigo de Miguel

**Affiliations:** † Department of Chemistry, NTNU—Norwegian University of Science and Technology, Trondheim NO-7491, Norway; ‡ Department of Teacher Education, NTNU—Norwegian University of Science and Technology, Trondheim NO-7491, Norway

## Abstract

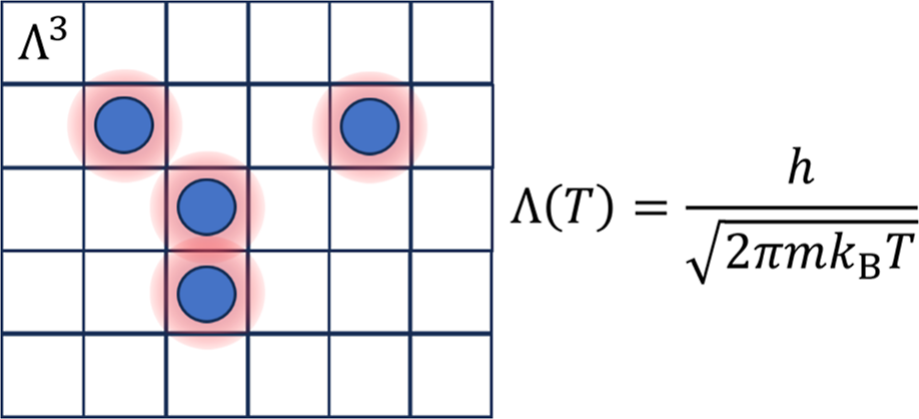

Lattice models are a central idea in statistical thermodynamics, as they allow for the introduction of a configurational entropy by counting the multiplicity of placing indistinguishable particles on a lattice. In this work we use a lattice model to show that the configurational entropy needs to be merged with the molecular translational entropy in order to have a consistent model. This is achieved by replacing the volume of a lattice site with a quantum volume (i.e., the cube of the thermal wavelength). This modified lattice model allows us to derive a new equation of state, referred to as the Bragg–Williams equation state, from which we may also derive a generalized version of the van der Waals equation of state. In contrast to the standard van der Waals equation of state, the heat capacity of the new models has temperature dependence.

## Introduction

Lattice models originated in the context of solid state physics, where the periodic pattern of crystal structures naturally resembles a lattice,^1^ and an early contribution using the multiplicity of placing molecules in different configurations is the classic work by Pauling on ice.^2^ In the broader context of physical chemistry, lattice models allow for a coarse-grained description of matter somewhere between the macroscopic realm of classical thermodynamics and an atomistic model of matter adopting quantum chemistry, and they may be useful to describe properties of polymers, alloys, fluids and gases (see e.g., refs (3–5)). Adopting the configurational entropy has, however, been questioned,^6^ with the argument that it implies, if not explained properly, that we have two distinct types of entropy, a “positional” entropy arising from the configurational entropy in addition to the entropy appearing in the second law. The concept of configurational entropy has therefore been the subject for further discussions.^7,8^ On the other hand, when interactions are considered, the positions of the particles need to be included in the entropy terms since interaction energies depend on the relative positions of the particles.^9^ We hope that this concern regarding the configurational entropy, which is a relevant point, is put at ease by this work, where the relation between the configurational and translational entropies is investigated.

The manuscript is organized as follows. We first discuss the quantum volume concept by considering the Helmholtz free energy of an ideal gas, followed by a demonstration of the relation between the configurational entropy in a lattice model and the translational part of the molecular partition function. We thereafter rederive the van der Waals (vdW) equation of state by adopting the Bragg–Williams approximation and by invoking the concept of quantum volume. We discuss the resulting modified equations of state as well as their corresponding Helmholtz free energies and partition functions. Finally, the internal energy and heat capacity for one of the new models are discussed. We close the paper with some concluding remarks.

## Helmholtz Free Energy of an Ideal Gas

The Helmholtz free energy, *F*, is for a monatomic ideal gas with *N* indistinguishable particles given by
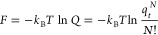
1where *T* is the temperature, *k*_B_ is Boltzmann’s constant, and *Q* is the partition function. Since electronic excitations for most molecules appear first in the UV/vis region, we regard in this work the electronic contribution to the molecular partition function, *q*_e_, to be independent of temperature, for example for closed-shell molecules *q*_e_ = 1 (for a detailed discussion of the molecular partition, see e.g. chapter 11 in ref (4)). For a monatomic ideal gas, the only contribution to the partition function arises thus from the translational part of the molecular partition function, *q*_t_, which is derived from quantum mechanics by considering a particle in a sufficiently large three-dimensional box at sufficiently high temperature, so that the energy levels may be treated as a continuum (see e.g. pages 250–253 in ref (10)), resulting in
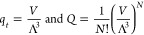
2where *V* is the volume of the system. The parameter Λ, sometimes called the thermal wavelength (see e.g. page 193 in ref (11), page 578 in ref (12)) or the quantum length (see page 253 in ref (10)), results from the partition function for a particle in a one-dimensional box in the high-temperature limit, and it is given by
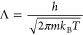
3where *m* is the mass of the atom and *h* is Planck’s constant.

The focus of this manuscript is to investigate a gas lattice model, and the contribution from a lattice model to the Helmholtz free energy, *F*_lattice_ = *U*_inter_ – *TS*_conf_, which for an ideal gas can be derived using a lattice model by simply placing *N* molecules on *M* lattice points. For an ideal gas, intermolecular interactions are ignored and therefore we set *U*_inter_ = 0 in the lattice model. All that remains is the configurational entropy, *S*_conf_, which is given in terms of the multiplicity, *W*, through Boltzmann’s entropy formula, *S*_conf_ = *k*_B_ ln *W*, with *W* given by the binomial distribution
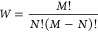
4i.e., the number of ways we can place *N* indistinguishable molecules on *M* indistinguishable lattice points. We adopt Maxwell–Boltzmann statistics for classical particles, and it is assumed that a lattice point is either empty or occupied by one particle. A justification for that also the empty lattice points should be regarded as indistinguishable is that then *W* = 1 for *N* = 0, resulting in that the “vacuum” free energy is zero for a lattice model. To obtain a result for an ideal gas and to demonstrate the resemblance with eq 1, the Helmholtz free energy is here derived in a somewhat nonstandard way as
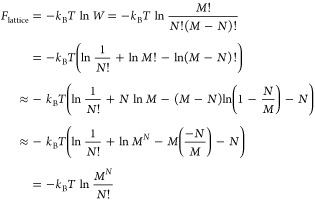
5where we first used Stirling’s approximation, ln  *M*! ≈ *M* ln *M* – *M*, and later *M* – *N* ≈ *M* as well as the first term in a Maclaurin series, ln(1 – *N*/*M*) ≈ −*N*/*M*, assuming that *M* ≫ *N*. The reason for not using Stirling’s approximation on the *N*! term, which would have been the standard approach, is to obtain an expression similar to eq 1.

Assuming that all lattice points have the same volume, *b*_0_, the total volume, *V*, is given as *V* = *b*_0_*M*. The lattice point volume is here denoted *b*_0_ since it is closely related to the excluded-volume parameter *b* in the vdW equation of state, which is discussed below. The Helmholtz free energy in eq 5 may thus be rewritten as
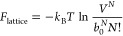
6

If we now adopt the concept by Yu,^13^ that *b*_0_ can be replaced by Λ^3^ where Λ is given in eq 3, we arrive at
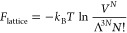
7and an identical expression to the Helmholtz free energy in eqs 1 and 2 has been obtained. The interpretation for a lattice model is that, in the limit of an ideal gas, there is an excluded volume, given by Λ^3^,^13^ that hinders two particles from occupying the same lattice point. To what extent this interpretation of Λ^3^ can be linked to a quantum mechanical picture, i.e., where two particles cannot occupy the same quantum state remains to be investigated.

The factor 1/Λ^3^ has been referred to as the quantum concentration (see page 74 in ref (14)) and Λ^3^ as the quantum volume,^10,13^ which is a term we also use here. However, quantum volume may be a misleading term since eq 3 does not imply in any way that the volume is quantized. Indeed, in the canonical ensemble, *V* is fixed, i.e., *V* is an independent variable and not dependent on *T*. Therefore, since *V* = Λ^3^*M* and Λ depends on the temperature, *M* must also have a smooth dependence on the temperature, which may be counterintuitive. In that sense, Λ^3^ may be regarded as a “statistical” volume rather than a physical volume, which is normally the way *b*_0_ is depicted in lattice models. Also note that the models discussed here for a given *V* are only valid for *N*Λ^3^/*V* < 1 since otherwise we would have more molecules than lattice points. A crucial difference between eqs 6 and 7 is that Λ, in contrast to *b*_0_, depends on the temperature, and consequently, the Sackur–Tetrode equation for the entropy of an ideal gas as well as *U* = 3*Nk*_B_*T*/2 are obtained from eq 7 but not from eq 6.^13^ It also implies that a particle momentum (kinetic energy) has been added to the model.

## Ideal Gas Law

In this section, we show that the idea of replacing the volume of a lattice point, *b*_0_, with Λ^3^ is not only an interesting concept,^13^ but that it actually is the only viable and even the only correct choice. A lattice model for a molecular system includes the interaction energies between the nearest-neighbor atoms, *U*_inter_, and also the configurational entropy for distributing the molecules on a lattice, *S*_conf_. One may argue that the intrinsic contribution to the Helmholtz free energy from the molecules given by the molecular partition function is missing and should also be added. It is thus tempting to regard the total Helmholtz free energy, *F*, as the sum of a contribution from the lattice model, *F*_lattice_ = *U*_inter_ – *TS*_conf_, and a contribution from the molecular partition function, , as
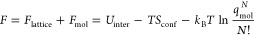
8where the notation *U*_inter_ and *S*_conf_ is used to emphasize that they do not denote the total internal energy and entropy, respectively, which also have contributions from *F*_mol_. The molecular partition function, *q*_mol_, may in turn be decomposed into partition functions for each degree of freedom as
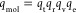
9i.e., a translational, rotational, vibrational and electronic contribution. For the molecular partition function, it is only the translational part, *q*_t_, given in eq 2, that depends on the volume and therefore contributes to the pressure.

If the pressure, *p*, is calculated from the Helmholtz free energy in eq 8, we get, however
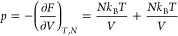
10i.e., twice the expected pressure for an ideal gas. One of the contributions arises from *F*_lattice_ given in eqs 6 or 7, which also has been derived in a different way (see pages 481–482 in ref (4)), and the other contribution arises from *q*_t_ in eqs 1 and 2. The only reasonable conclusion is that we (in error) have double counted in the sense that the same term has been included twice, and therefore that eq 7 is the only viable option for a lattice model so that it becomes identical to the translational contribution to the partition function in the limit of an ideal gas, i.e., *N*/*V* → 0.

To avoid the double counting of the term *q*_t_^*N*^/*N*!, the Helmholtz free energy for a lattice model in eq 8 should be modified as
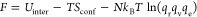
11we choose to keep *S*_conf_, rather than the *q*_t_^*N*^/*N*! in *F*_mol_, since *S*_conf_ is more general and it also includes nonideal contributions. As we shall see, this will allow us to derive the vdW equation of state by exploiting the approximations in the derivation of eq 5.

## Equations of State

The vdW equation of state is given as^15−17^
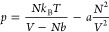
12where *a* > 0 and *b* > 0 are two (empirical) parameters where *a* accounts for attractive interactions between the molecules and *b* accounts for that a particle has a volume and is not treated as a point particle as is done in the ideal gas approximation.

The vdW equation of state is commonly derived in terms of an attractive 1/*R*^6^ interaction (see e.g., pages 286–289 in ref (18)), or from a system of hard spheres (see e.g., pages 304–305 in ref (19)). However, in his discussion of the Bragg–Williams (BW) approximation, Hill indicates that the vdW equation of state may also be derived from the BW approximation (see page 246 in ref (18)). Similar approaches are also discussed in early work by Heitler,^20^ and in an “alternative” derivation of the vdW equation of state by Reif (see pages 426–428 in ref (21)).

### Bragg–Williams Equation of State

The BW approximation is basically a mean field theory used in lattice models for solutions, whereby the probability to find a particle in a lattice point depends only on the “density” *N*/*M* and not on the interactions between the particles. Although this approximation was originally developed for alloys in materials science,^22−24^ it is also used in molecular thermodynamics as the basis to describe regular solutions, see e.g., chapter 15 in ref (4). In the following, we apply the BW approximation on a lattice model for a gas of *N* molecules distributed on *M* lattice points, i.e., with (*M* – *N*) empty lattice points.

For one molecule, the probability of finding another molecule on a neighboring site is, in line with the BW approximation
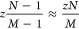
13where the coordination number *z* is the number of nearest-neighbor lattice points. With the pair interaction energy, *w* < 0, and repeating the procedure for all *N* molecules, the total interaction energy, *U*_inter_^BW^, becomes
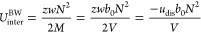
14where in the second step, *V* = *b*_0_*M*, and the factor 1/2 is included to avoid counting all the pair interactions twice. In the last step, we introduced an energy, *u*_dis_, which is the dissociation energy if all *z* nearest neighbor lattice points are occupied by a molecule
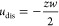
15where the factor 1/2 arises from the assumption that the binding energy between two particles is shared equally between both particles. The contribution to the pressure, *p*_U_^BW^, from the internal energy *U*_inter_^BW^ is calculated as
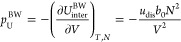
16resulting in the interaction term in the vdW equation of state in eq 12 with *a* ≡ *u*_dis_*b*_0_. The entropy from placing *N* particles on *M* lattice points is given by an intermediate result in eq 5 as
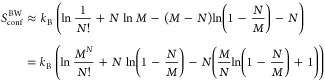
17

In the last term, it is noted that there is an exact cancellation of the *M*/*N* term by regarding the Maclaurin series of ln(1 – *x*) for (ln(1 – *x*)/*x* + 1), and the remaining terms behave as *N*/*M*. The entropy contribution to the pressure, *p*_S_^BW^, is consequently obtained as
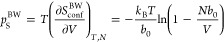
18where, again, *V* = *b*_0_*M*. Adding the two contributions to the pressure in eqs 16 and 18, we arrive at a pressure, *p*^BW^, that we here refer to as the BW equation of state
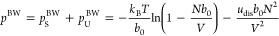
19

### Van der Waals Equation of State

To get from the BW equation of state in eq 19 to the vdW equation of state in eq 12, we follow the derivation of Dill and Bromberg (see pages 481–482 in ref (4)) and approximate *p*_*S*_^BW^ in eq 18 by first using the Maclaurin series ln(1 – *x*) ≈ −*x* – *x*^2^/2 followed by the approximation 1 + *x*/2 ≈ 1/(1 – *x*/2), leading to
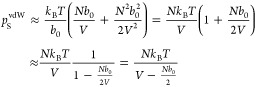
20which indeed is the entropy contribution to the vdW equation of state in eq 12 with *b* ≡ *b*_0_/2.

Since approximations beyond the BW model were used in the derivation of eq 20, we may regard the vdW equation state as an approximation of the BW equation of state. The vdW equation of state, as derived via the BW approximation and keeping the *b*_0_ and *u*_dis_ parameters in the equation, thus becomes
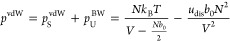
21We also note that, within our derivation from a lattice model, the *a* and *b* parameters in eq 12 are not independent since *a*/*b* = 2*u*_dis_.

### Quantum Volume Equations of State

Since Λ in eq 3 depends only on the temperature and not on the volume, we can follow the prescription in eq 7 and simply replace *b*_0_ by Λ^3^ in eqs 19 and 21, thereby obtaining two new equations of state with a different temperature dependence. We refer to these as the qv–vdW and the qv–BW equation of state, respectively, where *qv* denotes that the concept of quantum volume has been adopted. Thus
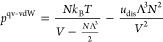
22and
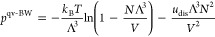
23

Note that *U*_inter_^BW^ in eq 14 is an approximation of the ensemble average of the interaction energy, not the microscopic interaction energy itself. Therefore, we should expect that *U*_inter_^BW^ depends on the temperature even if the interaction energies are independent of the temperature. This is fulfilled only in the qv-BW and qv-vdW equations of state in eqs 22 and 23, which result from invoking the temperature-dependent quantum volume. Furthermore, these equations of state have only one “free” parameter, the dissociation energy *u*_dis_, a parameter that has a clear physical interpretation, whereas the BW and vdW equations of state in eqs 19 and 21 have two parameters, *b*_0_ and *u*_dis_.

From eq 3, it is noted that Λ^3^ → 0 as *T* → ∞, and from that perspective, an atom is still treated as a point particle in the qv models, at least at high temperatures. It is, however, also noted that Λ = 0.7 Å for H_2_ at 300 K (and from eq 3, it will be even smaller for heavier molecules), which is around a factor of 5 smaller than typical intermolecular distances. Consequently, the lattice point volume arising from Λ^3^ is at least a factor of 100 smaller than the volume of a lattice point given by an empirical *b*-parameter. The repulsive part of the interaction energy, for example a hard sphere or the repulsive part of a Lennard-Jones potential, should therefore be included in the model for the interaction energy, *U*_inter_ (and not as an entropy term in *S*_conf_, in which we now replaced *b*_0_ by Λ^3^). In the BW approximation adopted here, *U*_inter_^BW^ in eq 14 is modeled by a single value, *u*_dis_, for the attractive part of the interaction energy. In a more sophisticated model, however, where *U*_inter_ includes also a short-range repulsive contribution to the interaction energy, there will be a competition between Λ(*T*) and *U*_inter_. A related discussion has been presented by Igolkin, where a modified translational partition function at high temperatures, including parameters from the interaction potential, is considered.^25^

Since Λ depends on the type of particle only through its mass, it would for molecular systems be natural to regard a model with one atom per lattice point so that a molecule extends over several lattice points,^26^ also in line with the Flory–Huggins model for polymers.^3^

### Comparison to Empirical Equations of State

Improvements beyond the vdW equation of state are mainly empirical where reproducing the critical point of the gas/fluid system has been central in the development of improved equations of state already from the start. Two very early modifications along these lines include Berthelot’s equation of state^27,28^
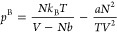
24

and the Dieterici equation of state^29^
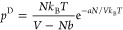
25both with a temperature dependence in the interaction term. In addition, Redlich and Kwong proposed an entirely empirical equation of state^30,31^
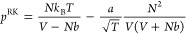
26where also here a notable feature is the appearance of a  dependence in the interaction term as compared to the vdW equation of state in eq 12. Important extensions of the Redlich–Kwong equation of state include the celebrated Soave–Redlich–Kwong,^32,33^ and Peng–Robinson equations of state.^34^

The temperature dependence adopted in these models are not identical, but they all to some extent resemble the 1/*T*^3/2^ dependence in the equations of state derived by including the concept of quantum volume in eqs 22 and 23. Although it is not our intention to compete with the wealth of empirical equations of state of great practical importance, many based on the Redlich–Kwong equation of state,^35,36^ the approach discussed here gives a theoretically sound motivation for a temperature dependence in the interaction term of an equation of state.

## Helmholtz Free Energies and Partition Functions

Given the Helmholtz free energy, or equivalently the partition function, all thermodynamic properties within the canonical ensemble can be obtained. In the following, we provide the Helmholtz free energy and the corresponding partition functions for the equations of state presented in eqs 19 and 21-23. The Helmholtz free energy for the BW model is given from eqs 14 and 17 as
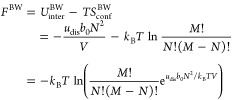
27

Hence, that the partition function for the BW approximation can be identified from *F* = −*k*_B_*T* ln *Q* as (see also page 247 in ref (18))
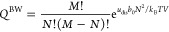
28

Adopting the approximate version of *S*_conf_^BW^ in eq 17, we have
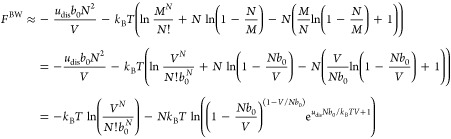
29again with *V* = *b*_0_*M*. This results in the following partition function
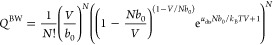
30

These latter two equations, eqs 29 and 30, are consistent with the here suggested BW equation of state in eq 19. The qv-BW model is obtained by simply replacing *b*_0_ with the temperature-dependent Λ^3^
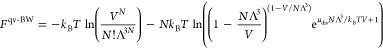
31and
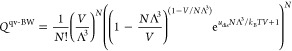
32which, as required, result in, respectively, the free energy in eq 1 and the partition function in eq 2 of an ideal gas in the limit *N*Λ^3^/*V* → 0. We also note that, even if interaction energies are ignored, *u*_dis_ = 0, we still have a nonideal correction that may be regarded as a (semiclassical) correction to the translational contribution and thereby to the kinetic energy.

The Helmholtz free energy corresponding to the vdW equation of state in eq 21 is less straightforward to obtain because of the approximations in eq 20, which were done to arrive at the prescribed equation of state through a lattice model. If we instead regard the vdW equation state as the starting point, the corresponding Helmholtz free energy is commonly obtained by integrating −*p*^vdW^*dV* to obtain *F* within a volume-independent function *f*(*T*, *N*), see e.g., ref (37)
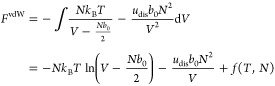
33

With the choice
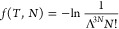
34the Helmholtz free energy corresponding to the vdW equation of state becomes, see e.g., page 174 in ref (38)
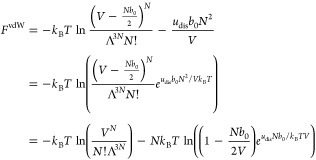
35so that the partition function is identified from *F* = −*k*_B_*T* ln *Q* as
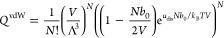
36We see that with the choice in eq 34, the Helmholtz free energy for an ideal gas in eq 7 is obtained in the limit *b*_0_ → 0, analogously to the choice to include the quantum volume in eqs 6 and 7. It is also noted, however, that in comparison to the lattice model investigated here, eq 35 is a kind of “halfway” approach where only one of the three *b*_0_-terms has been replaced by Λ^3^. For the qv-vdW model, we obtain
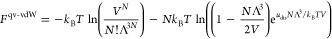
37and
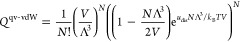
38by simply replacing *b*_0_ with Λ^3^ in the remaining two places. These two equations are entirely consistent with the qv–vdW equation of state in eq 22. Again, the free energy and the partition function can be phrased as the ideal contribution in eqs 1 and 2, respectively, followed by an interaction term that vanishes in the limit *N*Λ^3^/*V* → 0.

### Relation to Classical Partition Function

The classical partition function, *Q*_cl_, may be written as (see e.g. pages 113–117 in ref (19))
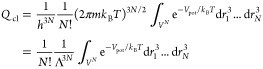
39where Λ was defined in eq 3, and *V*_pot_ is a potential energy in general dependent on all the coordinates in the system. The first part of eq 39 is given as a product of four factors, the last two accounting for kinetic and potential energy contributions, respectively. The potential energy factor is normally termed the configuration integral (it consists of 3*N* integrals involving the configurations, or positions, of *N* particles), and it becomes *V*^*N*^ if interactions are ignored, i.e. if *V*_pot_ = 0. The third factor is the contribution from the classical kinetic energy for *N* particles (it results from evaluating 3*N* momentum integrals). The second factor, 1/*N*!, accounts for the indistinguishability of identical particles and the first factor is 1/*h*^3*N*^ (for further details, we refer to the derivation by Hill, see pages 80–91 in ref (39)).

We note that for both the classical partition function and for the Helmholtz free energy corresponding to the vdW equation of state in eq 35, the quantum mechanical result is obtained in the limit of an ideal gas. This serves as support for our motivation for eq 7 that also the lattice model in terms of the configurational entropy should give the correct quantum result in the limit of an ideal gas.

### Internal Energy and Heat Capacity of the qv-vdW Model

For the remaining thermodynamic properties, we will restrict ourselves to the internal energy and heat capacity for the qv-vdW model (similar but not identical results are obtained for the qv-BW model). The reason is the well-known results for the vdW equation of state
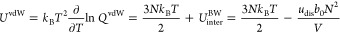
40and
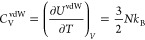
41which demonstrates a main deficiency of the vdW model, namely that *C*_V_, as for an ideal gas, is independent of the temperature. Due to the temperature-dependence of the quantum volume Λ^3^, two additional temperature dependencies are present in the nonideal part of the qv-vdW partition function in eq 38. This results in the following internal energy, *U*^qv-vdW^
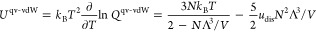
42which may also be expressed as
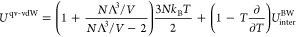
43where *U*_inter_^BW^, given by eq 14, becomes temperature dependent only when *b*_0_ is replaced with the quantum volume Λ^3^. The heat capacity, *C*_*V*_^qv-vdW^, becomes
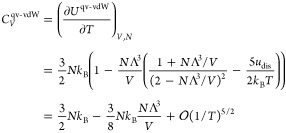
44A notable feature of the qv-vdW model is that, in contrast to the classical vdW model, the heat capacity is dependent on the temperature, and only in the high-temperature or low-density limit, the ideal temperature-independent heat capacity is recovered. The heat capacity has experimentally a divergence for the temperature at the triple point (see e.g., ref (40)), but we do not have a divergence in *C*_V_^qv-vdW^ because of the condition *N*Λ^3^/*V* < 1. Similarly, *C*_V_^qv-vdW^ > 0 at all temperatures as required.

## Concluding Remarks

The configurational entropy is a model entity, i.e., it is introduced in a lattice model but does not appear (explicitly) in other models. On the other hand, the kinetic energy (translational entropy) is a physical quantity that needs to be treated correctly in all models. By adopting the concept of quantum volume,^13^ we have demonstrated that the configurational entropy introduced in lattice models, in the limit of an ideal gas, corresponds to the quantum mechanical partition function for the translational motion, thereby merging the two contributions. Furthermore, the vdW equation of state has been rederived using the BW approximation resulting in the BW equation of state as a partial result. By invoking the quantum volume, two additional equations of state, qv-BW and qv-vdW, have been introduced, where a notable feature is that the heat capacity, *C*_V_, in contrast to the result for the vdW equation of state, has an expected dependence on the temperature.
